# Intralesional infiltration versus parenteral use of meglumine antimoniate for treatment of cutaneous leishmaniasis: A cost-effectiveness analysis

**DOI:** 10.1371/journal.pntd.0007856

**Published:** 2019-12-05

**Authors:** Nayara C. Brito, Tália S. Machado de Assis, Ana Rabello, Gláucia Cota

**Affiliations:** Pesquisa Clínica e Políticas Públicas em Doenças Infecto-Parasitárias-Centro de Pesquisas René Rachou-Fundação Oswaldo Cruz, Fiocruz, Belo Horizonte, Minas Gerais, Brazil; US Food and Drug Administration, UNITED STATES

## Abstract

Cutaneous leishmaniasis (LC) is a complex and variable disease in terms of epidemiology, aetiology, pathology and clinical characteristics. The mainstay of treatment is still pentavalent antimony (Sbv) compounds administered systemically, despite their recognized toxicity. The advantages of antimony intralesional (IL) infiltration are the use of lower doses of Sbv and, therefore, less toxic effects. The objective of this study was to estimate the cost-effectiveness ratio of intralesional meglumine antimoniate therapy (IL-MA) compared with endovenous meglumine antimoniate therapy (EV-MA) for the treatment of CL in the context of the Brazilian National Health System (SUS). An analytical decision model (decision tree) was developed using TreeAge Pro 2018 software. Data from the open-label, uncontrolled phase II clinical trial evaluating IL-MA were used as a reference for posology, efficacy, and adverse event rates (AE). The same premises for the intravenous approach (EV-MA) were extracted from systematic literature reviews. Macro and micro calculations of spending were included in the analysis. The IL-MA and EV-MA strategies had a total cost per patient cured of US$330.81 and US$494.16, respectively. The intralesional approach was dominant, meaning it was more economic and effective than was endovenous therapy. The incremental cost-effectiveness ratio showed that IL-MA could result in savings of US$864.37 for each additional patient cured, confirming that the IL-MA strategy is cost effective in the context of the Brazilian public health scenario.

## Author summary

Cutaneous leishmaniasis (LC) is a parasitic disease characterized by a non-fatal evolution but marked by significant morbidity resulting from lesions that are usually ulcerated, sometimes disfiguring, and distributed in exposed areas of the body. Pentavalent antimony compounds administered systemically (EV) are still considered the pillars of cutaneous leishmaniasis (CL) treatment despite its high toxicity. Other therapeutic approaches, including the intralesional infiltration of antimony derivatives have been proposed as an alternative as effective as the systemic route. In this study we performed a cost-effectiveness analysis of the meglumine antimoniate intralesional treatment (IL-MA) compared to systemic therapy with the same drug, from the perspective of the main source of pay in Brazil, Brazilian Public Health System (SUS). This is the first cost-effectiveness study evaluating the IL-MA approach compared to MA endovenously for the treatment of CL. Cost-effectiveness was expressed as the incremental costs per patient cured. In our model, IL-MA was more effective and led to substantially lower costs for the public health system. Analytical decision model showed that the IL-MA therapy could represent a cost saving of approximately US$ 877.64 per additional patient cured compared with the endovenous route, representing the more cost-effectiveness approach for treatment of selected CL cases in Brazil.

## Introduction

Leishmaniasis remains a public health problem in the Americas due to its clinical, biological and epidemiological complexity. In 2016, 17 endemic countries reported 48,915 cutaneous and mucosal leishmaniasis (CL/ML) cases to the Pan-American Health Organization (PAHO). The highest numbers were registered by Brazil (12,690), Colombia (10,966), Nicaragua (5,423) and Peru (7,271), which together are responsible for 74.3% of the total cases in the region. Among the 48,915 cases, 92 (0.19%) died. Analysis of leishmaniasis dataset of cases notified compulsorily to the sanitary authorities and registered in the Reportable Disease Information System (*Sistema de Informação de Agravos de Notificação*—SINAN, Brazilian Ministry of Health) has revealed an unexpected high death rate associated to CL/ML in recent years. Specifically, a lethality rate of 0.77 was observed between 2007 and 2014 for CL/ML, a rate much higher than that observed for dengue (0.09), which is considered a systemic and potentially lethal disease [1], [2]. This is an unexpected observation in view of the exclusive involvement of the integumentary system caused by the disease, which should not be a threat to life. On the other hand, considering the recognized toxicity observed with antimony derivatives, a drug used in more than 90% of treatments in Brazil, the relationship between deaths and adverse events secondary to the treatment emerges as a possible explanation for the observed lethality, a hypothesis still not definitely confirmed.

The anti-*Leishmania* therapeutic options are restricted and, around the world, antimonial derivatives are still the most widely used treatments for CL/ML, usually used parenterally and related to high toxicity. The most reported side effects are arthralgia, myalgia, loss of appetite, nausea, vomiting, abdominal pain, pruritus, fever, weakness, headache and dizziness. Associated with greater clinical significance, hepatitis, pancreatitis and electrocardiographic dose-dependent changes, such as ventricular repolarization abnormalities and QT interval prolongation, are well-described adverse effects. There are also cases of sudden cardiac death, probably related to ventricular arrhythmias [3].

The risk of developing ML, a complication caused by *Leishmania braziliensis*, is probably the main reason for the almost exclusively parenteral therapies used for CL in the Americas. This paradigm has been only recently overcome, which has allowed the accumulation of experience with topical and local treatments, including antimony intralesional infiltration [4], [5] [7]. Specifically, besides a systematic review gathering 5679 patients (global cure rate of 75%, IC 95% 68–81%), a single arm clinical study in Brazil has showed a cure rate of 87% (95% CI: 77–96%) and minimal systemic side effects with meglumine antimoniate intralesional infiltration approach [4], [5]. In 2010, the World Health Organization (WHO) recommended the inclusion of local and topical treatments among the acceptable therapeutic alternatives for CL in the Americas [8]. In 2013, PAHO also included in their regional guidelines antimony intralesional infiltration for selected cases [9]. From the economic point of view, it is a therapeutic modality that does not require investment in equipment, which makes its implementation feasible in the short term. However, to the best of our knowledge, a cost-effectiveness study evaluating this strategy has not been performed previously. Cost-effectiveness analyses in health describe interventions in terms of their cost per unit of health gain that they provide. It is a particularly important requirement for adequate allocation of resources in developing countries with severe limitations in the health budget. In addition, economic studies are a useful tool to guarantee access to effective technologies with equity by society, but but still little used in public policy decisions on neglected tropical diseases (NTD), including leishmaniasis [10].

The aim of this study was to estimate the cost-effectiveness ratio between meglumine antimoniate intralesional infiltration (IL-MA) and endovenous meglumine antimoniate therapy (EV-MA) for CL in the context of the Brazilian National Health System (SUS). This information is intended to guide healthcare providers in countries where CL is endemic.

## Methods

### Study design

The analysis to determine the cost-effectiveness ratio between IL-MA and EV-MA in a population affected by CL was performed through an analytical decision model (decision tree) using TreeAge Pro 2017 (TreeAge Software, Inc., Massachusetts, USA). The economic evaluation followed the Methodological Guideline: Economic Evaluation of Health Technologies of the Ministry of Health of Brazil [11].

### Input parameters

#### Therapeutic interventions

Two treatment regimens were compared: 1) N-methyl glucamine antimoniate endovenously at 15 mg per kg per day for 20 days, the standard therapeutic regimen recommended by the Brazilian leishmaniasis guidelines [6] as the first choice for patients younger than 50 years, non-pregnant and with no renal, hepatic or cardiac comorbidity; and 2) N-methyl glucamine antimoniate intralesionally, in weekly local infiltration, up to a maximum of eight infiltrations.

#### Effectiveness

The effectiveness parameter used to compare the interventions in this study was “cure rate”. Cure was considered the final outcome desirable after the treatment, defined as the complete re-epithelialization of all ulcerated lesions or a total involution of non-ulcerated lesions [12], in both cases accompanied by the complete regression of the infiltration and erythema, no relapse and no onset of any mucosal lesion at the 6-month follow-up.

#### Cure rate assumptions

The cure rate related to EV-MA treatment was obtained from a previously published systematic review [2], while the cure rate related to IL-MA was extracted from an open-label phase II clinical trial [4]. According to the systematic review performed by Tuon et al. (2008), the cure rate for the 310 Brazilian CL patients treated with MA by the parenteral route was 71.3% [13]. In this review, the authors included comparative and single arm studies, and *L*. *braziliensis* was the most prevalent species. In turn, for IL-MA approach, despite the existence of a systematic review gathering the studies addressing this therapeutic intervention in all world, few studies have been carried out in the Americas, with a great heterogeneity in term of procedure and posology. For this reason, considering the focus on the Brazilian scenario, we chose to use the cure rate observed in the only prospective study already published in Brazil, an open-label phase II clinical trial [4] which used a standardized therapeutic approach. In this study, 53 patients with confirmed CL presenting with a maximum of three skin lesions with a total area of less than 900 mm^2^ not involving ear, mucosal or joint regions were included. The MA intralesional infiltration was performed weekly using a validated technique. Overall, patients received a median of seven intralesional infiltrations over a median treatment period of 43 days. The definitive cure rate was 87% (95% CI:77–96%) [4].

#### Adverse event rate assumptions

The adverse event rate related to EV-MA treatment was obtained from a systematic review [10], a largest compilation of parenteral antimony toxicity data available, while the adverse event rate related to IL-MA was extracted from an open-label phase II clinical trial [4], the largest American prospective study designed to collect safety data for MA intralesional infiltration. For the two interventions, all adverse events reported in their respective sources with a frequency equal to or greater than 10% (very common) and need for some treatment or intervention were considered. For the EV-MA strategy, eight types of adverse events (clinical or laboratory) corresponding to adopted criteria were identified in the systematic revision performed by Oliveira et al., 2011 [3], which gathered 2900 patients treated with pentavalent antimonial parenterally. For the IL-MA, a strategy presenting significantly fewer adverse events, only five types of events were observed in a frequency equal to or greater than 10% in the phase II study [4] involving 53 patients that was used as source of this premise. Even so, we opted to include in the decision tree the eight events with the highest frequency also in this intervention.

#### Costs

Costs related to each treatment strategy included in this analysis were those represented by the drug itself, all medical supplies required for drug administration, professionals' fees and laboratory tests indicated for toxicity monitoring during the treatment period.

For estimating costs, an adult weighing 60 kg was considered the standard patient receiving EV-MA. As the volume of drug consumed in the intralesional infiltration treatment cannot be estimated from the patient’s characteristics, the median infiltrated volume per session reported in the phase II study was used here for the calculation of the intervention cost. Overall, patients received a median of seven infiltrations (IQR 25–75% 5–8) and a median total volume of 27.7 (16.4–46.4) ml of Glucantime over the course of the treatment [4].

Costs related to clinical and laboratory monitoring during the EV-MA approach were estimated based on the current recommendation of the Ministry of Health of Brazil, presented in a national guideline, which advocates the following weekly tests during the three weeks of treatment: electrocardiogram, blood count, amylase, lipase, urea, creatinine and liver enzyme levels [6]. No laboratory monitoring is recommended for patients during IL-MA treatment based on the absence of reports of systemic toxicity [3].

For both strategies, the salaries of the municipal health professionals of the municipality of Belo Horizonte, Minas Gerais, Brazil, were used as the reference. The drugs and medical supplies’ costs for each therapeutic strategy were obtained from the “Integrated System of Administration of General Services—SIASG” (available at: http://sigtap.datasus.gov.br/tabela-unificada/app/sec/inicio.jsp) and “Comprasnet” (available at: http://paineldeprecos.planejamento.gov.br/), two of the open information databases of the Brazilian public health system. The costs related to the laboratory monitoring during treatment, including electrocardiograms and blood tests, were obtained from the Brazilian Health System Reimbursement Values, a table containing values of exams and procedures paid for by the SUS [14].

The estimation of costs resulting from treatment toxicity considered the following rationale: conditions with available symptomatic treatment, such as pain, nausea, pruritus, the imputed cost is the cost of the generic symptomatic drug available in the Brazilian Health System and priced in SUS Reimbursement Values Table, at the maximum recommended dosage and throughout the duration of the treatment for leishmaniasis. For laboratory events, not accompanied by clinical manifestation, such as electrocardiographic abnormality, elevation of liver or pancreatic enzymes, it was considered for the cost estimation, the duplicate number of the respective monitoring tests to be performed throughout the treatment, a clinical approach routinely performed at Fiocruz Reference Center in Leishmaniasis, in Belo Horizonte, a unit specialized in the treatment of the disease in Brazil.

#### Cost-effectiveness analytical model

**Fig 1:**
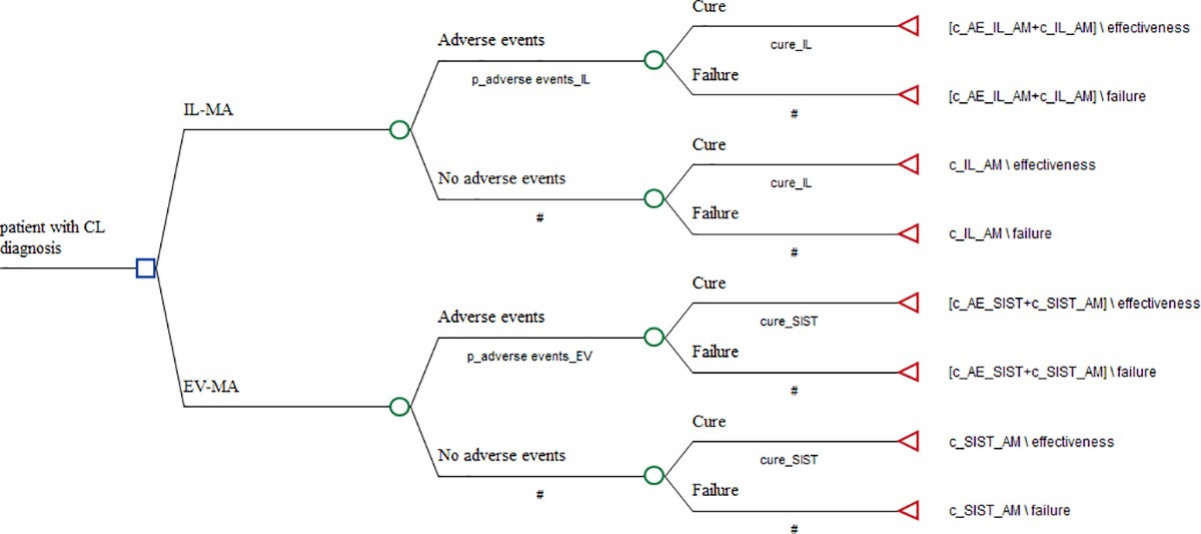
Simplified decision tree model for the analyses of the cost-effectiveness of intralesional infiltration, in comparison with parenteral use of meglumine antimoniate, in the treatment of cutaneous leishmaniasis. IL-MA: meglumine antimoniate intralesional infiltration; EV-MA: endovenous meglumine antimoniate therapy; p_adverse eventes_IL: probability of adverse events during IL-MA; p_adverse eventes_EV: probability of adverse events during during EV-MA; cure_SIST: cure rate for endovenous meglumine antimoniate therapy; cure_IL: cure rate for meglumine antimoniate intralesional infiltration; c_IL_AM: total cost for meglumine antimoniate intralesional infiltration; c_SIST_AM: total cost for endovenous meglumine antimoniate therapy; c_AE_IL_AM: total cost related to adverse events during IL-MA; c_AE_SIST: total cost related to adverse events during EV-MA.

A diagram with the variables used in the cost-effectiveness analytical model of therapeutic strategies for the treatment of cutaneous leishmaniasis is presented in Fig 1 and S1 Fig. The probabilities of events (adverse event and cure) were considered in a linear temporal perspective. The patient starts in the model with a confirmed diagnosis of CL and, and he is submitted to the probability of occurrence of each adverse event identified for each intervention, the patient may have none, one or more adverse events. After the occurrence of the adverse events, the hypothetical patient is submitted to the probability of cure, according to the cure rates adopted for each intervention. The result is determined in a binary outcome as cure or therapeutic failure. Failed patients are not retreated.

The analytical perspective was that adopted by the Brazilian public health system—*Sistema Único de Saúde* (SUS)—i.e., the payers’ perspective, and the time horizon was six months, a set point currently accepted as the appropriate time for CL definitive cure assessment. For this study, only direct medical-hospital costs were considered at a macro and micro level. For all costs, it was considered the values in force in 2018.

### Cost-effectiveness analysis

The cost-effectiveness ratio was expressed in terms of the cost per patient cured in both therapeutic interventions. The incremental cost-effectiveness ratio (ICER) was used to estimate the cost per additional patient cured after IL-MA compared to the EV-MA therapy. In other words, the ICER represents the difference in magnitude between the two alternatives using data retrieved from a clinical trial and a systematic review, expressed in terms of the incremental cost per unit of effect, as follows [11]:

$$\text{Incremental}\;\text{Cost}\;\text{Effectiveness}\;\text{Ratio}\;(\text{ICER})=\frac{\Delta \;\text{C}}{\Delta \;\text{E}}=\frac{\left(\text{Costs}\;\text{EV-MA})\text{-}(\text{Costs}\;\text{IL-MA}\right)}{\left(\text{EV-MA}\;\text{effectiveness-IL})\text{-}\;(\text{IL-MA}\;\text{effectiveness}\right)}$$

EV_MA: endovenous meglumine antimoniate treatment; IL-MA: intralesional meglumine antimoniate therapy

All comparisons were explored in a sensitivity analysis in order to identify parameters influencing the results and to estimate the threshold values above or below which one strategy becomes preferred over the other. In Brazil, there is no threshold definition for a cost-effective intervention and the traditional 1–3 times the gross domestic product (GDP) per capita, proposed by WHO [15], is considered an high and unfordable amount for developing countries. Alternatively, and considered more appropriate to the Brazilian scenario, we adopted the threshold proposed by Pichon-Rivière et al. (2017), who estimated the threshold for Brazil as follows: an ICER of 0.66 GDP per capita or less is considered “cost-effective”, less than 0.32 is “very cost-effective” and greater than 0.99 is "non-cost-effective" [16]. The Brazilian GDP/per capita in 2016, calculated at US$8,639.36 [17], was used as the reference value in this analysis.

For the cost-effectiveness analysis (CEA), the costs were estimated and expressed in real (R$, Brazilian currency) and converted into US dollars. The effectiveness was expressed in terms of the cure rate, and the ICER was expressed in cost per patient cured. The exchange rate between the real and the US dollar was US$1 = R$3.7158 on July 29, 2018.

### Sensitivity analysis

In order to test the uncertainty derived from the components of the decision model as well as the reliability and robustness of the cost-effectiveness analysis, a deterministic one-way sensitivity analysis was conducted. In this analysis, isolated variations in critical measures are imposed on the model in order to verify changes in the final ICER.

Probabilistic sensitivity analysis was performed using a Monte Carlo simulation, including microsimulation of 10,000 people, and the acceptability curve for different thresholds for the willingness to pay. The probabilistic analysis provides a distribution of ICERs from which cost-effectiveness acceptability curves can be derived. The parameters included in the probabilistic sensitivity analysis were cure rate and costs. For sensitivity analysis, it was assumed that probabilities have a β (beta) distribution in a narrow range from 0 to 1, whereas costs, follows a γ (gamma) distribution that can take any value greater than zero. For IL-MA efficacy rate, the 95% confidence interval (95% CI) reported by Ramalho et al. (87% - 95% CI:77–96%) [3] and the overall cure rate reported in the systematic review performed by Brito el al. [5] were used as parameters in the sensitivity analysis. For EV-MA, the efficacy rate was varied arbitrarily by ±20% considering the lack of the confidence interval in the original study. The costs related to the drugs, medical procedures and laboratory tests were varied arbitrarily by ± 25%. Finally, a tornado diagram was used to illustrate the one-way analysis and the acceptability curve of cost-effectiveness in the probabilistic sensitivity analysis.

## Results

### The treatment costs

The estimated direct costs of the EV-MA and IL-MA strategies were US$457.50 and US$330.76, respectively. In addition, the costs related to monitoring and treating AEs were estimated at US$98.5 and US$4.85 for the EV-MA and IL-MA groups, respectively (Tables 1 and 2).

**Table 1:** The costs related to meglumine antimoniate intralesional infiltration and to endovenous meglumine antimoniate treatment.

Description	Estimated value (US$)
**Meglumine antimoniate by the endovenous route (EV-MA)**	
**Medical doctor’s salary (five doctor's appointments)**	130.06
Nurse’s salary (20 nurse appointments)	221.31
Glucantime: total of 40 ampoules	50.37
Supplies for AM administration	22.87
Supplies for blood collection for laboratory tests	1.77
Electrocardiogram	5.52
Blood count test	4.41
**Biochemical tests**	
Urea	1.98
Creatinine	1.98
Alkaline phosphatase	2.16
Aspartate aminotransferase (AST)	2.16
Alanine aminotransferase (ALT)	2.16
Gamma-glutamyl-transferase (GAMA GT)	3.77
Amylase	2.41
bilirubin	2.16
Lipase	2.41
**Total**	**457.50**
**Meglumine antimoniate intralesional infiltration (IL-MA)**	
Medical doctor’s salary (7 doctor appointments to perform the intralesional infiltration)	182.98
Nurse’s salary	134.27
Glucantime, total of 7 ampoules	8.81
Lidocaine hydrochloride 2% solution for local anaesthesia	0.69
AM infiltration supplies	
Syringes: 12 units	0.90
Hypodermic needle: 24 units	0.44
Chlorhexidine gluconate solution: 30 ml	0.15
Sodium chloride 0.9%: 30 ml	0.05
Sterile gauze pad: 120 units	0.68
Gloves	0.41
Surgical gloves	1.37
**Total**	**330.76**

**Table 2:** The costs related to adverse events to meglumine antimoniate intralesional infiltration and to endovenous meglumine antimoniate treatment.

Adverse events	Probability of occurrence	Adverse event’s treatment or triggered action	Estimated cost (US$)
Endovenous meglumine antimoniate therapy*			
Pain in drug administration site	0.64	analgesic	1.23
↑ Lipase / amylase	0.60	monitoring of lipase / amylase levels performed more often (twice a week)	21.19
Myalgia / arthralgia	0.49	analgesic	1.23
↑ AST / ALT	0.43	monitoring of AST / ALT levels performed more often (twice a week)	21.19
Ventricular repolarization changes	0.25	electrocardiogram performed more often (twice a week)	22.10
Headache	0.24	Analgesic	1.23
Fever	0.17	Antipyretic	1.23
QTc interval prolongation	0.16	electrocardiogram performed more often (twice a week)	22.10
**Total**			**91.50**
**Meglumine antimoniate intralesional infiltration** **			
Itching	0.79	antihistamine drug	0.34
Pain in drug administration site	0.34	analgesic drug	0.43
Erythema	0.32	antihistamine drug	0.34
Myalgia	0.16	analgesic drug	0.43
Secondary infection	0.13	antibiotic	2.03
Joint pain	0.10	analgesic drug	0.43
Headache	0.05	analgesic drug	0.43
Fever	0.05	antipyretic drug	0.43
**Total**			**4.85**

*Oliveira et al. (2011)

**Ramalho et al. (2018)

AST: aspartate transaminase alanine transaminase (ALT)

The EV-AM approach adds costs related to toxicity monitoring on the order of 7%. In addition to these costs, a further 21% is required for the treatment of adverse events. On the other hand, 96% of the costs of the IL-MA are due to the salaries of the medical professionals responsible for the infiltration procedure and only 1% of the total costs are related to monitoring and treatment of AEs. The most frequently reported clinical adverse events with EV-MA, reported by Oliveira et al., 2011[3] were pain at the drug administration site, myalgia, arthralgia, headache and fever. Also based on this systematic review, the laboratory abnormalities most frequently described were a mild to moderate increase of aspartate transaminase (AST), alanine transaminase (ALT), lipase and amylase levels, besides the prolongation of the QTc interval and ventricular repolarization disorders in the electrocardiographic monitoring (Table 2). Among patients undergoing intralesional therapy, the incidence of AEs was significantly lower; the predominant symptoms were itching, pain at the drug administration site, erythema, myalgia, secondary infection, headache and fever (Table 2).

### Cost-effectiveness ratio and sensitivity analysis

The decision tree developed for evaluating the cost-effectiveness of IL-MA compared to EV-MA for CL treatment in Brazil (costs per cure) is shown in Fig 1.

The IL-MA resulted in a total cost per patient treated and cured of US$330.81, while EV-MA resulted in a cost of US$494.16 per patient cured, which implies an incremental cost of US$163 with the second option. IL-MA was more economic and effective than EV-MA, with an incremental efficiency of 0.186, which characterizes a relation of domination. The incremental cost-effectiveness ratio showed that IL-MA would result in savings of US$864.37 for each additional patient cured (Table 3).

**Table 3:** Cost-effectiveness analysis of meglumine antimoniate intralesional infiltration compared to endovenous meglumine antimoniate for cutaneous leishmaniasis treatment in Brazil.

Strategy	Cost	Incremental cost	Effectiveness	Incremental effectiveness	ICER	Dominance
**EV-MA**	494.16		0,684			dominated
**IL-MA**	330.81	-163.34	0.870	0.186	-864.37	undominated

ICER. Incremental cost effectiveness EV-MA: meglumine antimoniate administered endovenously

IL-MA: meglumine antimoniate intralesional infiltration

In addition, by using the cure rate reported in a systematic review performed by Brito et al., 2018, a negative result for the incremental cost-effectiveness ratio for IL-MA *versus* EV-MA was observed, confirming the dominance status of the IL_MA (ICER: - 1835, 77).

**Fig 2:**
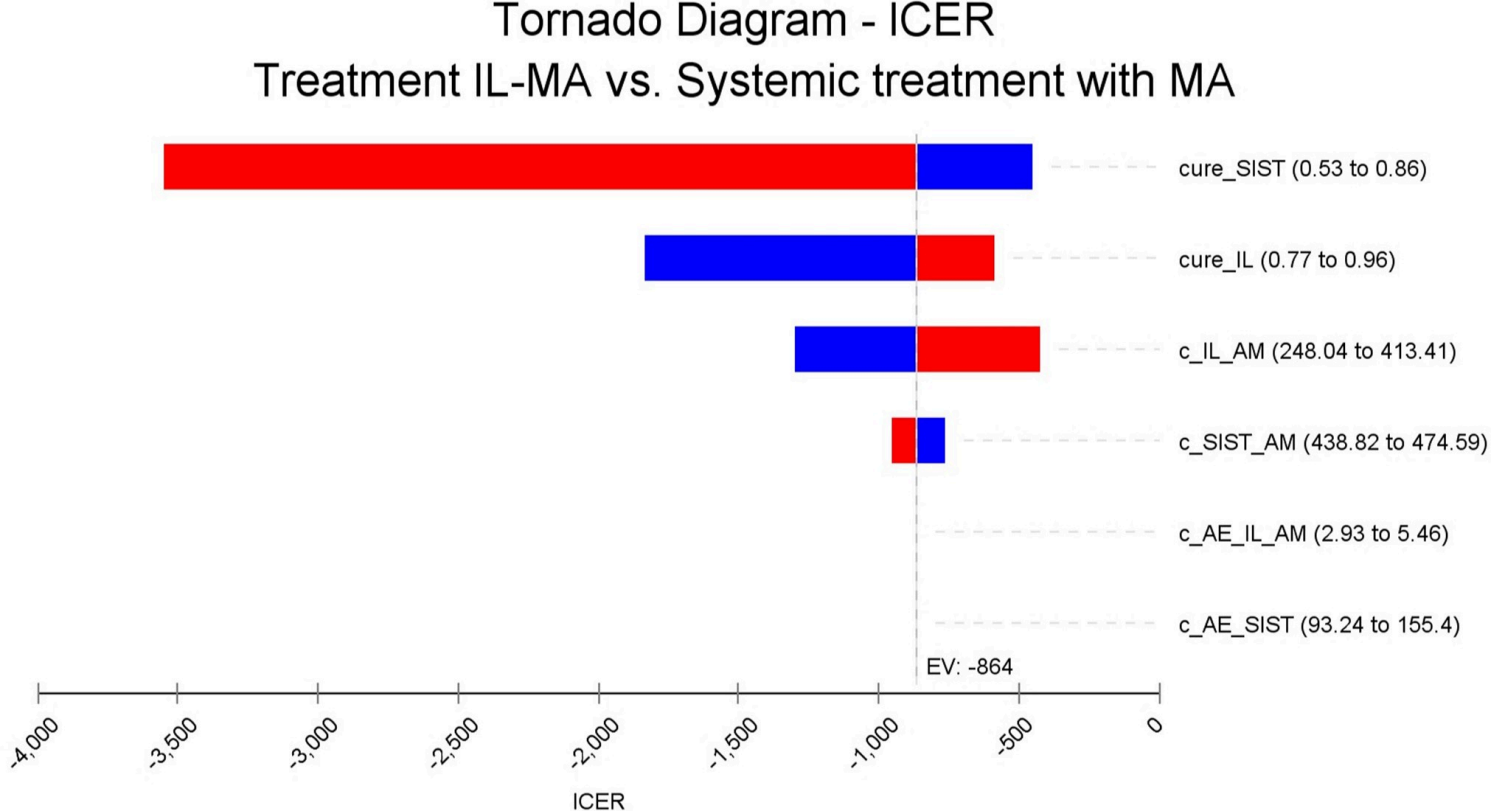
Tornado diagram of deterministic sensitivity analysis. **Notes:** Vertical line represents final ICER. Critical variables are represented as horizontal bars. All variables crossed final ICER, which means that none of them was able to affect the result. cure_SIST: cure rate for endovenous meglumine antimoniate therapy; cure_IL: cure rate for meglumine antimoniate intralesional infiltration; c_IL_AM: total cost for meglumine antimoniate intralesional infiltration; c_SIST_AM: total cost for endovenous meglumine antimoniate therapy; c_AE_IL_AM: total cost related to adverse events during IL-MA; c_AE_SIST: total cost related to adverse events during EV-MA.

The deterministic sensitivity analysis, using costs and efficacies parameters variation, revealed the EV-MA cure rate as the parameter that incorporated the greater uncertainty in the incremental cost-effectiveness ratio (Fig 2). It is worth stating that IL-MA remains cost-effective in all scenarios explored in this study, having as threshold 0.66 GDP per capita. Even considering a higher cure rate for EV-MA (86%), the IL-MA approach would still save US$541.68 per additional cure; that is, even in the worse scenario, IL-MA is highly cost-effective (Fig 2).

**Fig 3:**
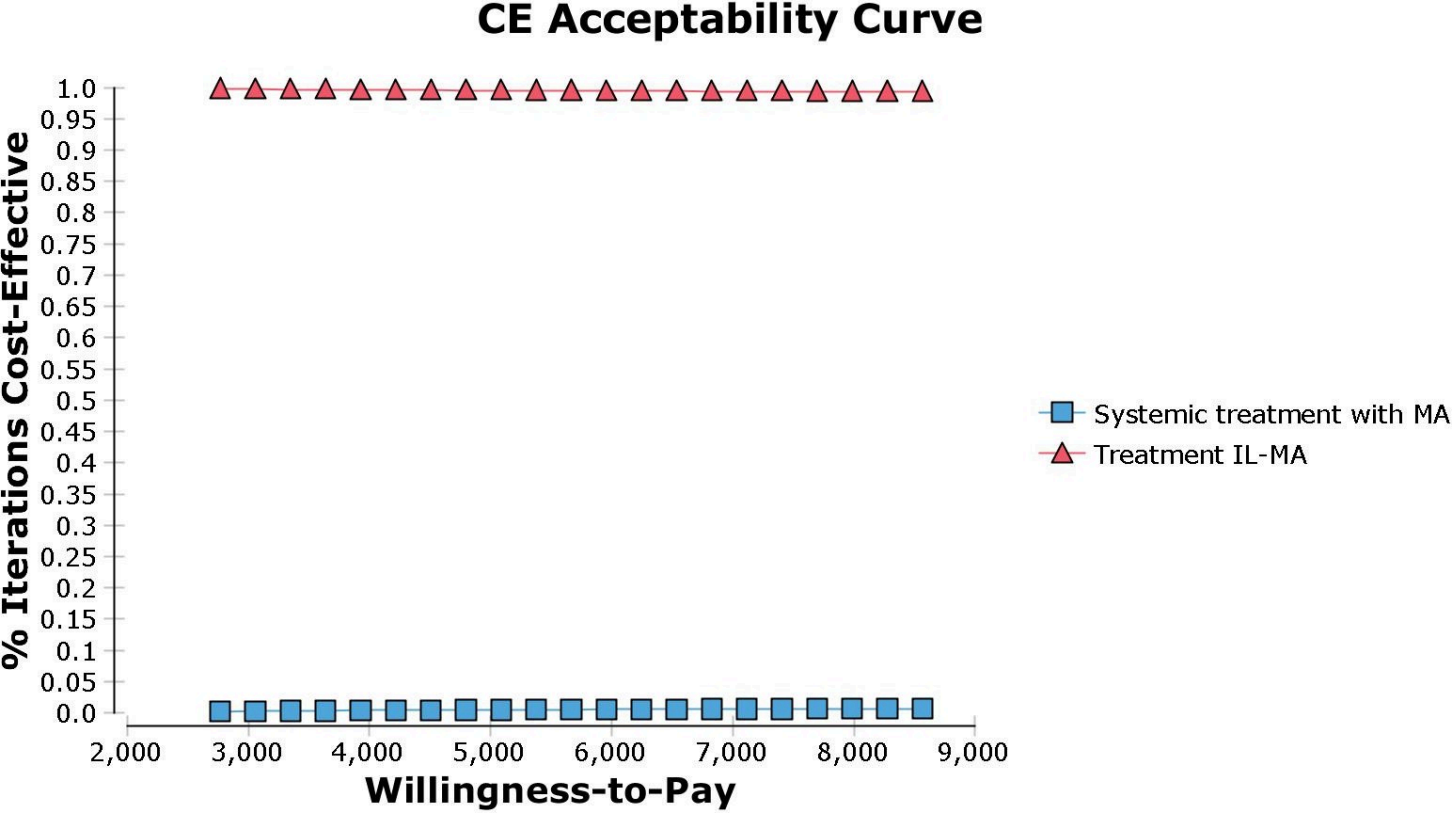
Cost-effectiveness acceptability curve. MA: meglumine antimoniate; IL-MA: meglumine antimoniate intralesional infiltration.

The cost-effectiveness acceptability curve was obtained from the Monte Carlo simulation. Cost and cure rate parameters were varied into the model (Fig 3). The graph shows the probability of a given treatment be the most cost-effective alternative for a range of willingness-to-pay (WTP) values. By varying the WTP value from US$2767.98 to US$8563.45, the probability of IL-MA remains the most cost-effective treatment varies from 99.7% to 99.3%, respectively, which means that it is the dominant strategy in all scenarios. In conclusion, the sensitivity analysis indicated that the study finding is robust under variations in effectiveness, safety, and costs parameters.

## Discussion

This is the first cost-effectiveness study comparing IL-MA and EV-MA strategies for CL treatment in the New World. An analytical decision model showed that the IL-MA approach saves costs from the perspective of the Brazilian public health system compared to the parenteral treatment with the same drug. These results are comprehensive because they include costs related to the treatment of adverse events and are robust, withstanding variations of various parameters, including efficacy and costs. The evidence presented here supports the implementation of IL-MA therapy for patients with localized CL [3]–[5] in the context of the Brazilian public health system.

There are only two studies addressing the antimony intralesional approach concerning to cost-effectiveness perspective. Both studies were carried out in Afghanistan using sodium stibogluconate (SSG) as the comparator. Specifically, the cost-effectiveness analysis performed by Reithinger et al. showed that the Afghan standard treatment (intralesional and intramuscular administration of antimonial) costs US$27 (95% CI: 20–36) per patient cured and US$ 1200 (95% CI  =  761–1827) per disability adjusted life year (DALY) averted [18]. According to the authors, an intramuscular SSG treatment would cost US$3,718 per DALY averted. Analyses of the cost-effectiveness of the antimony intralesional approach alone were not presented. In addition, Stahl et al. evaluated the cost-effectiveness of simple wound debridement and moist wound treatment, compared to intralesional sodium stibogluconate (IL-SSG) from a societal perspective. Average costs per patient were US$11 for IL-SSG. When compared to IL-Sbv, the ICER for the wound debridement regimens were US$0.09 and US$0.77 per day, respectively, per wound free day [19].

Refai and collaborators evaluated the efficacy, safety, and cost-effectiveness of thermotherapy in a non-inferiority randomized blinded controlled trial. In this study, patients were randomly assigned to a single session of radiofrequency-induced heat therapy (RFHT) or to IL-SSG. The costs were collected from a social perspective, and effectiveness was measured in injury-free days. The RFHT strategy costs were seven times lower (USD = 1.54/patient) than those related to the IL-SSG (US$11.09/patient); however, the cost-effectiveness rate was not presented [20], hampering direct comparison with our findings. It is important to note that the studies conducted in Afghanistan and on the Indian subcontinent differ significantly in several aspects from the study conducted in Brazil. New and Old World CL are diseases with distinct clinical presentations and natural evolution, as expected considering the different species of *Leishmania* involved. Moreover, from the methodological point of view, the studies used very different strategies to compare the therapeutic interventions, to collect costs and to present the results. An evident difference can be observed in the treatment costs charged in Asian studies, significantly lower than those reported here and in other American studies. This could be explained by the low cost of drugs in Asian countries compared to the cost of drugs in South America due to differences in drug purchasing price (e.g., use of generic *versus* branded drugs), therapy protocol and health professional salaries. More importantly, costs related to adverse events were not included in all of these prior studies. All these aspects must justify the large discrepancy in costs between MA intralesional infiltration estimated by us in Brazil (US$50.37) and SSG intralesional infiltration in Afghanistan (US$1.13), both quoted in US dollars.

There are few studies addressing the cost-effectiveness of CL therapies in the New World. Thermotherapy, another local therapy for CL, was evaluated in two cost-effectiveness studies [21], [22] in comparison to antimony parenteral treatment. In the study conducted by Cardona-Arias et al., the costs generated by parenteral MA therapy and thermotherapy were estimated to be US$66,807 and US$14,079, respectively. The average cost-effectiveness ratios were US$632.4 (ranging from US$590 to US$721 according to the sensitivity analysis) for MA and US$298.4 (ranging from US$246 to US$352) for thermotherapy, and the incremental cost-effectiveness ratio for each cured patient was US$2,523 (ranging from US$2,323 to US$4,073), confirming thermotherapy’s dominance [22]. In another study carried out by the same authors, addressing the cost-effectiveness of thermotherapy *versus* MA in any administration route, antimony therapy generated a total cost of US$2,731,276 plus US$58,254 to cover the treatment of adverse events. MA had an estimated total of US$4241 per DALY averted and US$85 per patient cured [21]. All these evidences contribute to the consolidation of local therapies, such as antimony intralesional infiltration and thermotherapy, as more cost-effective alternatives for CL treatment in Americas, in comparison to parenteral administration of antimony derivatives. It is also evident that a great part of this superiority is due to the reduction of the costs with monitoring and treatment of adverse events, more frequent with antimony parenteral therapy. There are, however, significant differences between the local approaches, for example, unlike intralesional infiltration, thermotherapy requires initial investment with equipment acquisition.

The main limitation of this current cost-effectiveness study was not to include the perspective of society in the accounting of costs, that is, expenses paid by patients and their families related to transportation and loss of working days, among others. In addition, the use of data extracted from a clinical study may also be considered a limitation, although while increasing the rate of identification of adverse events and allowing the detailed measurement of the procedures, it may reflect a reality different from the daily routine of health services. Moreover, as in any economic study, extrapolation of these results requires caution considering that the costs of acquiring medicines and reimbursing health professionals may vary significantly among different countries and even within the same country. Other intrinsic aspects in different scenarios also need to be considered prior to the implementation of the conclusions observed here, such as the organization of health services in remote areas, especially those related to the legal and technical competences of the professional assigned to carry out the intralesional infiltration. Despite these limitations, this study has strengths such as the inclusion of costs related to therapy toxicity and not just the costs related to the treatment itself; in addition, the adverse event rates were obtained from a clinical study (active observation) or from a systematic review (a comprehensive source), which reduces the risk of underestimation of event occurrence rates. Also favouring its reliability, we have considered the amounts paid for by the public health system in Brazil for the estimation of costs.

Until now, a threshold of cost-effectiveness has not been established in Brazil, despite the requirement of a cut-off for any decision to incorporate a new health technology by the public health system. In 2001, the WHO Macroeconomics Commission proposed that technologies that prevent one disability-adjusted life year (DALY) for less than 3 GDP per capita should be considered cost-effective and those whose cost is less than 1 GDP per capita, very cost-effective [15]. However, the lack of specificity of this threshold has already been widely recognized and admitted by WHO [23], which now recommends that each country set the threshold most appropriate to its reality. There are many reasons to consider a universal threshold as an inadequate definition, among them the existence of countries with high GDP per capita that allocate small proportion of its budget in health. In addition, the WHO threshold is considered high for most countries [24], which makes most interventions eligible to be incorporated, an impracticable task due to the limited resources budgets. Therefore, if averted DALYs are more highly valued in high-income countries, the use of cost–effectiveness thresholds based on per capita GDP per DALY averted will give a biased measure of the willingness to pay [24]. Alternatively, in this study, we considered the threshold estimated by Pichon-Rivière, a definition of threshold based on the relationship between per capita budget in health and life expectancy, which ultimately incorporates some of the characteristics of each country. According to this proposal, the cost-effectiveness threshold for Brazil should be between 0.62–1.05 GDP per capita/ for quality adjusted life years (QALY). It is important to note that, regardless of the definition of the ideal threshold for Brazil, in the present IL-MA *versus* EV-MA case, in all scenarios, the IL-MA remains dominant, that is, cost-effective.

Cutaneous leishmaniasis is a high burden disease in Brazil with a significant part of its morbidity related to standard treatment with antimonial derivatives that are highly toxic, thus requiring frequent laboratory monitoring that generates even more costs for the public health system. In this scenario, the main advantage of the intralesional MA approach, including the economic perspective, is a significant decrease in the serious adverse events rate.

In conclusion, IL-MA therapy could represent a cost saving of approximately US$864.37 per additional patient cured compared with the same drug endovenously administered. Our findings complement previous studies addressing efficacy and confirms that IL-MA is a cost-effective strategy for CL treatment from the perspective of Brazil's public health system. The cost-effectiveness ratio provides fundamental information to guide health managers, and the evidence generated in this study can be useful for guiding recommendations about CL, properly allocating limited resources and improving the efficiency of the public health system.

## Supporting information

S1 Fig Decision tree model for the analyses of the cost-effectiveness of intralesional infiltration, in comparison with parenteral use of meglumine antimoniate, in the treatment of cutaneous leishmaniasis. IL-MA: meglumine antimoniate intralesional infiltration; EV-MA: endovenous meglumine antimoniate therapy; c_AE_IL_AM: total cost related to adverse events during IL-MA; c_AE_SIST: total cost related to adverse events during EV-MA; c_art_IL: total cost of joint pain treatment; c_AST_ALT: total cost of monitoring aspartate transaminase and alanine transaminase; c_erythema_IL: total cost of treatment of erythema patient during IL-MA; c_fever_IL: total cost of treating a patient with fever during IL-MA; c_fever_SIST: total cost of treating a patient with fever during EV-MA; c_headache_IL: total cost of treating headache patients during IL-MA; c_headache_SIST: total cost of treating headache patients during EV-MA; c_IL_AM: total cost for meglumine antimoniate intralesional infiltration; c_infec_sec_IL: total cost of treatment of a patient with secondary infection during IL-MA; c_itching: total cost of patient treatment itching during IL-MA; c_lipase_amylase: total cost of monitoring lipase and amylase enzymes; c_myalgia: total cost of treatment of patients with myalgia during IL-MA; c_myalgia_SIST: total cost of treatment of patients with myalgia during EV-MA; c_pain_IL: total cost of treating patients with pain in drug administration site during IL-MA; c_pain_SIST: total cost of treating patients with pain in drug administration site during EV-MA; c_QTc: total cost of monitoring patients with QTc interval prolongation; c_SIST_AM: total cost for endovenous meglumine antimoniate therapy; c_VR: total cost of monitoring patients with ventricular repolarization changes; cure_IL: cure rate for meglumine antimoniate intralesional infiltration; cure_SIST: cure rate for endovenous meglumine antimoniate therapy; p_art_IL: probability of joint pain during IL-MA; p_AST_ALT: probability of change in the levels of aspartate transaminase and alanine transaminase enzymes; p_erythema_IL: probability of erythema during IL-MA; p_fever_IL: probability of fever during IL-MA; p_fever_SIST: probability of fever during EV-MA; p_headache_IL: probability of headache during IL-MA; p_headache_SIST: probability of headache during EV-MA; p_infec_sec_IL: probability of secondary infection during IL-MA; p_itching_IL: probability of itching during IL-MA; p_Lipase_amylase: probability of change in the levels of lipase and amylase enzymes; p_myalgia_IL: probability of myalgia during IL-MA; p_myalgia_SIST: probability of myalgia during EV-MA; p_pain_IL: probability of pain in drug administration site during IL-MA; p_pain_SIST: probability of pain in drug administration site during EV-MA; p_QT_SIST: probability of QTc interval prolongation; p_VR_SIST: probability of ventricular repolarization changes.
(ZIP)
